# Woman

**Published:** 1908-03

**Authors:** 


**Woman. A Treatise on the Normal and Pathological Emotions of Feminine Love.** By **Bernard S. Talmey, M. D.,** Gynecologist to the Metropolitan Hospital and Dispensary, New York. 12mo, pp. 300. Illustrated. New York: Stanley Press Co. (Price, $3.00.)

When Dr. Talmey took up this subject for the making of a book, even a small one, he entered a field which, while not wholly virgin, is at least fertile; and he showed more than mere literary courage. Briefly, the work is a treatise on the normal and pathologic emotions of love in the woman, and it is doubtful if there are many of the phases of the sexual life or thoughts of woman which the author has not snatched from their hiding places and laid upon the altar of his literary ambition. It takes a brave man to do such deeds as these and, although he is wise enough to not lay claim to having disclosed all that women think of in the realm of love and sexuality—God forbid that anyone should attempt so impossible a task—yet he does sort of set himself up, by virtue of priority, as a pioneer.

It is true, as pointed out by the author, that the physiology of the sexual emotions is not taught in the medical schools; that is, not as a part of the curriculum sanctioned by the faculty.

It is equally true that women suffering from disturbances of sexual emotions do not find physicians capable of giving them relief; at least, not always. These considerations led Dr. Talmey to write the book, and all other questions aside it is a distinct addition to medical literature, for medical literature should be complete; and how can it be when it is so barren of instruction regarding the sexual thought and emotions of women? There has been some criticism of the book on the ground that it is objectionable in character. That is unfair; for after all the book is merely theoretic, and being so it may possibly be made valuable if the public could be brought to pluck its wisdom in such matters from the pages of a book rather than spend time in the pursuits of clinical experience. But it is doubtful if even so well written a book as Dr. Talmey's,—and it is well written,—will ever become a fixture in scientific literature. The analysis of woman and her thoughts and her sexual impulses is not a science; it is an art.

